# Sub1 and Maf1, Two Effectors of RNA Polymerase III, Are Involved in the Yeast Quiescence Cycle

**DOI:** 10.1371/journal.pone.0114587

**Published:** 2014-12-22

**Authors:** Joël Acker, Ngoc-Thuy-Trinh Nguyen, Marie Vandamme, Arounie Tavenet, Audrey Briand-Suleau, Christine Conesa

**Affiliations:** 1 iBiTec-S CEA, FRE3377, Gif-sur-Yvette, France; 2 CNRS, FRE3377, Gif-sur-Yvette, France; 3 Université Paris-Sud, FRE3377, Gif-sur-Yvette, France; Newcastle University, United Kingdom

## Abstract

Sub1 and Maf1 exert an opposite effect on RNA polymerase III transcription interfering with different steps of the transcription cycle. In this study, we present evidence that Sub1 and Maf1 also exhibit an opposite role on yeast chronological life span. First, cells lacking Sub1 need more time than wild type to exit from resting and this lag in re-proliferation is correlated with a delay in transcriptional reactivation. Second, our data show that the capacity of the cells to properly establish a quiescent state is impaired in the absence of Sub1 resulting in a premature death that is dependent on the Ras/PKA and Tor1/Sch9 signalling pathways. On the other hand, we show that *maf1Δ* cells are long-lived mutant suggesting a connection between Pol III transcription and yeast longevity.

## Introduction

In its natural environment, the budding yeast *Saccharomyces cerevisiae* spends most of its life in a quiescent state where it can maintain both viability and the capacity to re-enter the proliferation cycle upon the addition of a carbon source. The length of time a non-dividing population of cells maintains viability defines its chronological life span (CLS). For years now, yeast CLS provides a fruitful model for aging research and many progress have been made to understand the mechanisms regulating the cell quiescence cycle, including the entry into quiescence, the maintenance of viability and the capacity to re-proliferate. A number of dramatic morphological, physiological and biochemical changes [1], [2] allows the cells to properly establish a resting state. A massive reprogramming of gene expression occurs with a down regulation of most of the genes transcribed by all three RNA polymerases. These changes are under the control of the Ras/PKA and Tor1/Sch9 signalling pathways that integrate nutrient availability to regulate cell proliferation. To repress transcription by RNA polymerase (Pol) III, these pathways converge to Maf1 [3], [4]. Upon starvation, Maf1 is dephosphorylated and accumulates in the nucleus where it interacts directly with the Pol III transcription machinery, leading to transcriptional repression [5].

In contrast to Maf1 whose function is essentially to integrate responses from signalling pathways to regulate Pol III transcription, Sub1 and its human ortholog PC4 are involved in distinct DNA-dependent processes including replication, DNA repair and transcription [6]. Sub1 has multiple roles in different steps of the Pol II [7], [8] and Pol III transcription cycle. In vitro, Sub1 stimulates both Pol III transcription initiation and reinitiation [9]. In vivo, the inactivation of *SUB1* correlates with a decrease in Pol III transcription efficiency without affecting the cell growth rate in exponential phase. Genome wide occupancy mapping reveals that Sub1 is present on all Pol III-transcribed genes, on the rDNA gene locus (note however that Sub1 binding to the 35S rRNA gene is controversial [9], [10]) and on a subset of highly Pol II-transcribed genes. Whether or not the binding of Sub1 to Pol II-transcribed genes correlates with a role in their transcription is not known yet, with the exception of some individual genes like *IMD2* [11] or *HSP26* [10]. Interestingly, Sub1 occupies all the genes related to the protein synthesis machinery (ribosomal RNAs, tRNAs and ribosomal protein genes) whose transcription accounts for 80% of nuclear transcription in proliferating cells and that are strongly repressed in stationary phase.

We analysed here the possible role of Sub1 in reactivating transcription when cells are exiting stationary phase and begin to re-proliferate. To better understand the relationships between Sub1 and Maf1 that exert an opposite effect on Pol III transcription, we carried out our experiments not only in strains deprived of Sub1 or Maf1 but also in a mutant in which both genes were deleted. Our experiments show that the relationships between Maf1 and Sub1 are more complex than anticipated and do not correspond to classical synthetic genetic interactions. In the present study, we provide several lines of evidence that Sub1 and Maf1 are involved in different steps of the quiescence cycle and have an opposite effect on yeast chronological life span.

## Materials and Methods

### Yeast strains and plasmids

Standard techniques were used for cloning of plasmids and transformation of yeast cells. All strains used in this study are derivatives of strain YPH500. A first set of strains (Set 1) consists of null derivatives [9], [12] of Sub1 (*sub1Δ::kanMX6*) or Maf1 (*maf1Δ::ura3*) that were combined to generate *sub1Δmaf1Δ* mutant Unless otherwise indicated, this set of strains was used for experiments. Null derivatives of Sub1 and/or Maf1 were also generated using *sub1Δ::his3* and/or *maf1Δ::kanMX6* deletion cassettes (Set 2). The *atg1*, *ras2*, *rim15*, *sch9* or *sod2* null derivatives were generated by transformation of YPH500 or su*b1Δ::his3* strains with PCR-amplified *kanMX4* cassettes from the corresponding null strains of the Yeast Knockout Collection (Open Biosystems). The C160-13myc strains were generated using *RPC160-13Myc::kanMX6* and *sub1Δ::his3* cassettes. The Sub1-13myc strain was generated using *SUB1-13Myc::kanMX6* epitope-tagging cassette. Since uracil auxotrophy affects longevity [13], the strains used for experiments performed in SC or SD4x medium were all uracil prototrophs either because of the deleting cassette or by transformation with a *URA3*-marked plasmid.

For the construction of a plasmid overexpressing *SOD2*, a 1600-bp genomic fragment encompassing the *SOD2* locus was amplified by PCR from yeast genomic DNA and cloned into the *URA3*-marked pRS424 plasmid yielding pRS424-SOD2. A≈3-fold increase in Sod2 protein levels was measured by western blot analysis in cells transformed with pRS424-SOD2 plasmid.

### Growth conditions

Media and techniques are described in Sherman (1991) [14]. Nutrient-rich yeast extract/peptone/dextrose medium (YPD) was supplemented with adenine for most of the experiments to prevent the strong red colour (due to the *ade2-101* mutation of our background strain) of cultures grown to stationary phase that may interfere with some experiments such as microarray hybridization. Synthetic complete (SC) glucose containing medium was supplemented with adenine and all amino acids for which the strains were auxotrophic at a concentration (1 x) routinely described in the literature [14]. Synthetic dextrose was supplemented 4-fold (SD4x) with adenine and the amino acids for which the strains were auxotrophic. No uracil was added in SC or SD4x medium since all strains were uracil prototrophs. Only freshly prepared liquid medium was used since we observed that the survival of *sub1Δ* or *sub1Δmaf1Δ* strains was reduced in SC medium older than one week. Starter cultures were used to inoculate appropriate volumes of fresh medium to an optical density (OD_600_) of 0.1. When OD_600_ = 0.8–1 is reached, the time point is designated as day0 or exponential phase. Starter cultures were inoculated only from fresh plates since we observed that when cultures were inoculated from plates that had been left at room temperature or at 4°C for a few days, the *sub1Δmaf1Δ* strain did not systematically regrow. After 24 h, the titers of cultures reproducibly differed from strain to strain, the *maf1Δ* strain and strains lacking Sub1 reaching a higher or lower OD_600_ than wild type, respectively.

### Chronological life span

Viability was determined over time using trypan blue exclusion assay, quantitative plating experiments or serial dilutions. For trypan blue staining, 100 µl of cultures were centrifugated and cells were resuspended in 20 µl of trypan blue 0.4%. After 5 min at room temperature, images were taken randomly using a Leica DMRXA microscope and the ratio of viable to inviable cells was determined by counting black or white cells. For quantitative plating experiments, 10 ml of cultures were centrifugated at day2 and cells were resuspended into the same volume of water to avoid regrowth of viable cells. Samples of cultures were serially diluted in YPD then spread onto YPD plates. For spotting assays, 10-fold serial dilutions were made in YPD and 4 µl was spotted onto YPD plates.

### Flow cytometry

Flow cytometry measurements of DNA content were performed as described [15]. Briefly, 2 OD_600_ of cells taken at each time point were pelleted by centrifugation, washed with water and resuspended in 70% ethanol. After one night at 4°C, the pellets were washed with water, treated with 2 mg/ml of RNase A (Sigma) for 2 h at 37°C, then with 5 mg/ml of pepsin (Sigma) for 20 min at 37°C. The pellets were resuspended in 500 µl of 50 mM Tris-HCl pH 7.5, and 50 µl of cells were stained by adding 500 µl of 50 mM Tris-HCl pH 7.5 containing 1 µM SytoxGreen (Invitrogen). For DHE staining, 2 OD_600_ of cells were pelleted by centrifugation, resuspended in 100 µl phosphate-buffered saline (PBS) containing 50 µM dihydroethidium (DHE, Molecular Probes) and incubated at 30°C for 10 min. Cells were then pelleted, washed with PBS and resuspended in 1 ml PBS. After a brief sonication, flow cytometry was carried out on a FACSCalibur model flow cytometer (BD Biosciences) and data were processed using CellQuest software (BD Biosciences). A total of 25,000 cells were analysed for each curve. The percentage of cells in each phase was determined using the ModFit LT 4.0 cell-cycle modelling software (Verity). For DHE staining, the y axis shows the number of cells measured at a given point on the x axis which is a logarithmic scale of arbitrary fluorescence units.

### RNA analyses

For RNA analyses, total RNA was isolated by using the hot phenol acid extraction protocol as described [9]. tRNA^Ileu^ and tRNA^Leu^ genes were chosen for Northern and RT-qPCR experiments since they have been previously used in works studying Pol III transcription in the absence of Maf1 or Sub1 [9], [12].

#### Metabolic labeling

Day4 stationary phase cells grown in SD4x medium supplemented with appropriate components were used to inoculate appropriate volumes of fresh medium to OD_600_ = 0.5. At each time point, total RNAs were labelled for 10 min by adding 150 µCi of (5,6 ^3^H)-uracil (1 mCi/ml) to 10 ml of cell cultures as described previously [9]. Three µg of total RNA were analysed by gel electrophoresis under denaturating conditions (acrylamide 8%, urea 7 M, TBE 1x). The gel was stained with ethidium bromide, processed for fluorography with Amplify (GE Healthcare) and then dried before autoradiography. Two independent in vivo labelling experiments were performed and found to give reproducible results.

#### Northern blot

Twenty µg of total RNA per lane was loaded onto a denaturing gel. After blotting, the membranes were hybridized with the appropriate ^32^P-labeled probes. The oligonucleotide probes were 5′-TGCTCGAGGTGGGGATTGAACCCACGACGGTC for Pre-tRNA^Ileu^ and tRNA^Ileu^ and 5′-GATTGCAGCACCTGAGTTTCG for 5S rRNA.

#### RT-qPCR

First-strand cDNA for each sample was synthesized by reverse transcription (RT) of 3 µg of total RNA, using SuperScript II reverse transcriptase (Invitrogen) and random primers (Invitrogen) according to the manufacturer's instructions. There is a large excess of the mature as compared to the pre-tRNAs in total RNA preparations. We thus analysed by PCR the products obtained after RT reactions using primers designed to amplify both mature and intron-containing forms of tRNAs. Only the larger DNA fragment corresponding to the intron-containing form was observed for the tRNA^Ileu^, tRNA^Leu^ or tRNA^Pro^ amplifications suggesting that the reverse transcriptase enzyme was not able to synthetize cDNA from the mature form of tRNAs, probably because of the numerous modifications occurring on tRNAs or their highly stable tri-dimensional structures that might resist heat denaturation. Thus, only pre-tRNAs are represented after RT reactions. Real-time PCR was performed using an ABI Prism 7300 machine (Applied Biosystems). The PCR reactions were carried out in 20 µl containing 0.4 µM each primer and 10 µl of mastermix SYBR green PCR reaction (Applied Biosystems). All sets of reactions were conducted in triplicate or more on at least two independent preparations of cDNA.

The primers were 5′-GCGCCGTGGCGCAGTGGAAGCGCG and 5′-GCGCCGCTCGGTTTCGATCCGAGGAC for Pre-tRNA^iMet^, 5′-GGTTGTTTGGCCGAGCG and 5′-TATTCCCACAGTTAACTGCGGTCA for Pre-tRNA^Leu^, 5′-GCTCGTGTAGCTCAGTG and 5′-TCGTTTTAAAGGCCTGT for Pre-tRNA^Ileu^, 5′-GTTGCGGCCATATCTACCAGA and 5′-TCGCGTATGGTCACCCACTAC for 5S rRNA, 5′-TCACGGAATGGTACGTTTGA and 5′-GCGAAGGATTTGGTGGATT for 35S rRNA, 5'-GTTGGTCCAGATTTGGCTGT and 5′-ACGAACGTCATCTTCCTTGG for *RPS6A*, 5′-CATGTTCCCAGGTATTGCCGA and 5′-GTCAAAGAAGCCAAGATAGAA for *ACT1*, 5′-CCAAGAGCCTACCAAGACCG and 5′-CGCAAAATCGACTAAACCTGG for *IMD2*, 5′-TCGGTATCGTTGTTCCAAGA and 5′-CGAATTCACCCAACAAAGTG for *SSB1*, 5′-AAGTCGTGGTTCCTGGTGTC and 5′-TGTCAAAACACCATTTGCGT for *HSP26*.

#### Microarray hybridization

Hybridizations on Pol III-specific microarrays were performed and quantified as described [12]. Briefly, total RNA (20 µg) supplemented with control RNAs used for normalization (CAB, RCA, and RbCl from Stratagene) was reverse-transcribed using a mixture of Pol III-specific primers and poly(dT) oligonucleotide. As described above, only neo-synthetized tRNAs levels were analysed using microarrays since mature tRNAs could not be reverse-transcribed under our conditions.

### Protein analyses

Protein analyses were performed essentially as described [5]. Proteins extracted using trichloroacetic acid and acid-washed glass beads were analyzed by SDS-PAGE and Western blotting. Analysis of Maf1 levels by immunoblotting was performed using a modified acrylamide∶bisacrylamide ratio (33.5∶0.3) SDS-PAGE. For co-immunoprecipitation experiments, protein extracts were incubated with magnetic beads coated with anti-myc antibodies. The beads were washed, and the bound polypeptides were eluted and analyzed by SDS-PAGE, followed by immunoblotting using anti-myc or anti-Maf1 antibodies. Quantification was performed using Quantity One software (Bio-Rad).

### Fluorescence microscopy

Sub1-13myc cells grown to exponential or stationary phase (day5) in YPD were fixed for 30 min in formaldehyde (3.7%). 9E10 anti-myc antibodies or polyclonal antibodies directed against the A190 subunit of Pol I or Maf1 were used to detect Sub1, Pol I or Maf1, respectively. The secondary antibodies were either Alexa fluor 594 (red) or 488 (green) antibodies (Invitrogen). Cells were counterstained with DAPI (4′,6′-diamidino-2-phenylindole). All observations were made with a Leica DMRXA microscope equipped with a Roper Scientific MicroMax cooled charge-coupled device camera and MetaMorph software (Molecular Devices).

## Results

### Sub1 is important for survival and optimal re-proliferation of quiescent cells

During the course of our experiments on the regulation of Pol III transcription, we reproducibly observed that cells deprived of Sub1 needed more time than wild type to regrow when inoculated into fresh liquid medium. These growth defects were exacerbated in a double *sub1Δmaf1Δ* mutant which prompted us to study more closely the role of Sub1 and Maf1 in the quiescent cycle.

**Figure 1:**
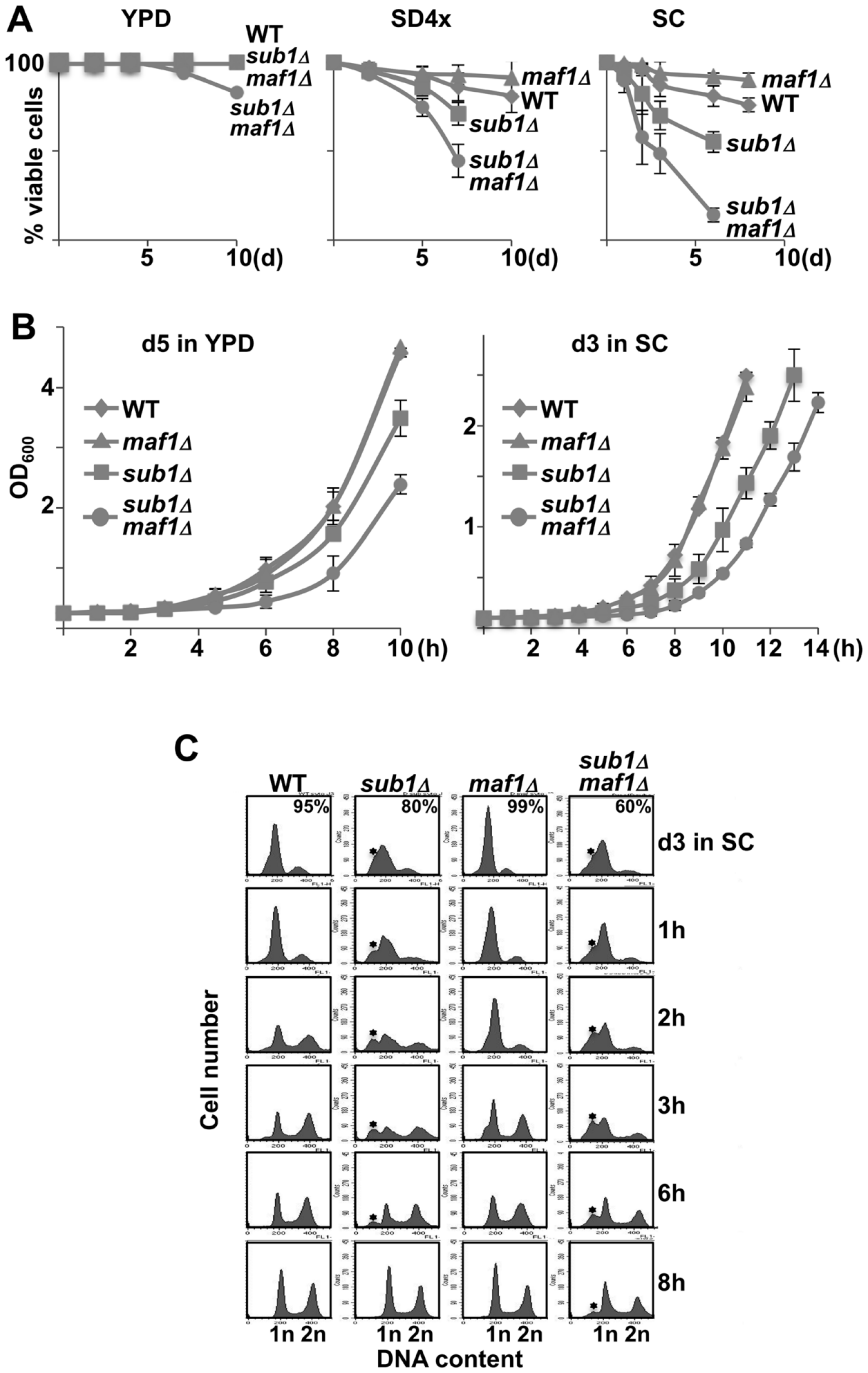
Loss of Sub1 results in a premature death and a delay to exit from resting. (**A**) Viability of the indicated strains (all uracil prototrophs, see Materials and Methods) grown in YPD, SD4x or SC medium was determined over time by trypan blue exclusion assays. Viability curves represent means and standard deviations from at least three independent experiments. (**B**) The same number of viable cells determined by trypan blue staining of day5 or day3 samples in YPD or SC medium, respectively, was re-inoculated into fresh media. Optical density at 600 nm was monitored over time as a measure of regrowth. The standard deviations were calculated from at least three independent experiments. (**C**) Day3 cultures in SC medium were re-inoculated into fresh media and DNA content was determined over time by flow cytometry. The percentage of viable cells in the resting cultures is indicated. The star indicates the position of sub-G1 peaks corresponding to apoptotic DNA degradation. The percentage of cells in each phase was determined using the cell cycle modelling software ModFit LT 4.0 and is shown in S2 Fig.

Since a delay in exiting quiescence could be due to a loss of cell viability, the proportion of living cells was measured over time using trypan blue staining of cells grown to stationary phase in nutrient-rich YPD medium, in synthetic complete (SC) medium or in SD medium supplemented 4X with the appropriate components (SD4x). A differential cell viability loss was indeed observed depending on the medium (Fig. 1A). In YPD, no cell viability loss could be observed except for the *sub1Δmaf1Δ* strain at day10. In both SD4x and SC, cells deprived of Sub1 were defective in maintaining viability under non-growth conditions i.e displayed a shortened chronological life span (CLS). In contrast, *maf1Δ* cells displayed an extended CLS as compared to the wild type strain. This opposite role of Sub1 and Maf1 on the CLS is explored below.

To assess whether Sub1 and Maf1 could play a role in the re-proliferation of quiescent cells, we monitored growth curves starting from the same number of viable cells as measured by trypan blue staining. Resting cells at day5 in YPD (no mortality, see Fig. 1A) or day3 in SC (≈25% and ≈50% mortality for *sub1Δ* or *sub1Δmaf1Δ* cells, respectively) were inoculated into fresh media and the OD_600_ was monitored over time as a measure of re-growth. All the strains were able to re-enter the proliferation state (Fig. 1B). However, a reproducible delay was observed in both media in cells lacking Sub1 suggesting that these cells needed more time to exit from resting state. Once in exponential phase, the doubling time of wild type and mutant strains were indistinguishable. A similar delay in resting exit was also observed by monitoring growth curves in SD4x, with our second set of mutant strains generated in YPH500 background with different deleting cassettes and with a *sub1Δ* strain generated in BY4741 background (S1 Fig.) indicating that this phenotype was not a yeast background dependent effect.

In order to use another approach, DNA content was analysed by flow cytometry in resting cells and after their release into fresh SC medium. The percentage of cells in each phase is shown in S2 Fig. The presence of dead cells in *sub1Δ* or *sub1Δmaf1Δ* cultures at day3 in SC was correlated to the presence of sub-G1 peaks (Fig. 1C, star) corresponding to apoptotic DNA degradation [16]. Cell cycle progression from resting to mitotic cycling as visualized by the relative proportion of 1n or 2n DNA peaks was much slower in *sub1Δ* and *sub1Δmaf1Δ* cells as compared to wild type. Interestingly, a≈1 h delay in restarting proliferation, probably not long enough to be observed by monitoring growth curves (Fig. 1B) could be visualized in *maf1Δ* cells (Fig. 1C and S2 Fig., lanes 2h). Note also that *maf1Δ*cells arrested more efficiently with a G1 content of DNA than wild type cells (Fig. 1C and S2 Fig., lanes d3). Whether this more efficient G1 arrest of *maf1Δ*cells could be correlated to their ≈1 h delay in restarting proliferation needs to be further investigated. Our data showed that cells deprived of Sub1 were delayed in exiting quiescence in all experimental conditions we assayed and that this phenotype was not dependent on viability loss. The defects of *sub1Δ* cells in resting exit were exacerbated in the absence of Maf1 suggesting synthetic sick genetic interactions between *SUB1* and *MAF1* genes. An additive effect of Sub1 and Maf1 deletions may explain the longer delay in quiescence exit observed for the *sub1Δmaf1Δ* double mutant.

### Sub1 is involved in the reactivation of transcription upon exit from resting

**Figure 2:**
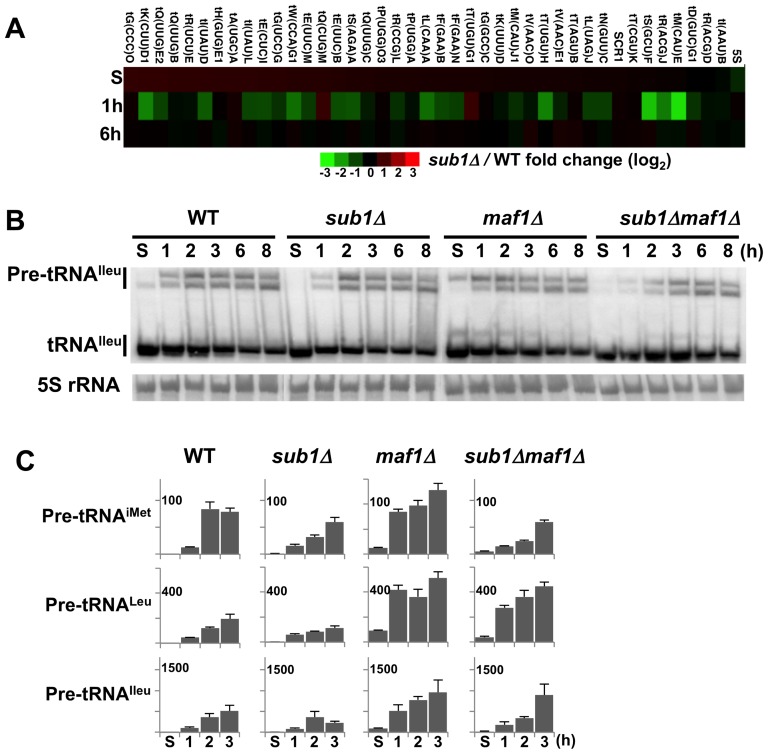
Sub1 is required for an optimal reactivation of Pol III transcription when cells are exiting quiescence. (**A**) Microarray hybridization. Expression ratios of a subset of Pol III-transcribed genes in the *sub1Δ* mutant and the wild type strain grown in YPD, at day10 stationary phase (S) and after 1 or 6 h post-inoculation into fresh medium are shown. Results for the indicated genes are presented according to the red-green color scale. (**B**) Northern blot analysis. Total RNA isolated from the indicated strains grown in YPD, at day5 (S) or after 1 to 8 h post-inoculation into fresh medium was hybridized with probes designed to reveal both pre-tRNA^Ileu^ and tRNA^Ileu^, or 5S rRNA. (**C**) Real-time PCR analysis. The expression of tDNA^iMet^, tDNA^Leu^ or tDNA^Ileu^ genes in the indicated strains grown in YPD at day5 (S) or after 1 to 3 h post-inoculation into fresh medium was determined by real-time PCR. Relative gene expression levels were calculated using 5S rRNA as an internal control since its levels did not significantly change over time in all strains. Standard deviations were calculated from two independent preparations of cDNA.

Since its deletion correlates with a delay in exiting quiescence (Fig. 1), we wondered whether Sub1 could play a role in the rapid reactivation of transcription occurring upon exit from resting. To prevent any misinterpretation of these transcription studies due to the presence of dead cells in quiescent cultures, we performed all our experiments in YPD or SD4x at days where no significant cell viability loss could be observed. The level of Pol III transcription was first analysed in wild type and *sub1Δ*strains using a Pol III-specific microarray [12]. First-strand cDNAs were synthesized by reverse transcription of total RNA isolated from resting cells in YPD and 1 h or 6 h after their inoculation into fresh medium. As described in Materials and Methods, only pre-tRNAs species could be reverse-transcribed under our experimental conditions. The gene array shown on Fig. 2A displays the *sub1Δ*/WT normalized ratio of each probe according to the red-green colour-scale. A slight decrease in the global amounts of neo-synthetized tRNAs could be observed at 1 h post-inoculation (mean of all ratios of 0.74). After 6 h, the transcriptional profiles of both strains were indistinguishable (mean of all ratios of 1.04). However as already observed for Maf1 regulation of tRNA gene expression using similar microarrays [12], not all genes were regulated to a similar extent. More than half of the genes were less induced in *sub1Δ* cells at 1 h post-inoculation (lane 1h, green) showing that Sub1 was necessary to reactivate transcription of these genes at an early stage of the exit from resting. The most delayed gene was *IMT4* encoding tRNA^iMet^ (7 fold lower). This gene, as well as the one encoding tRNA^Ileu^ (2.5 fold lower) or tRNA^Leu^ (3 fold lower) were chosen for further analysis by Northern (Fig. 2B) or quantitative PCR (qRT–PCR) (Fig. 2C). Both experiments confirmed that in the absence of Sub1, the reactivation of Pol III transcription was delayed and/or lower than in wild type. In contrast, Maf1 deletion could lead as expected to a Pol III transcription reactivation occurring faster and to a greater extent than in wild type. Strikingly, the deletion of Maf1 in *sub1Δ* cells resulted in a more complex pattern of reactivation, revealing both transcriptional delay and/or higher transcriptional levels.

**Figure 3:**
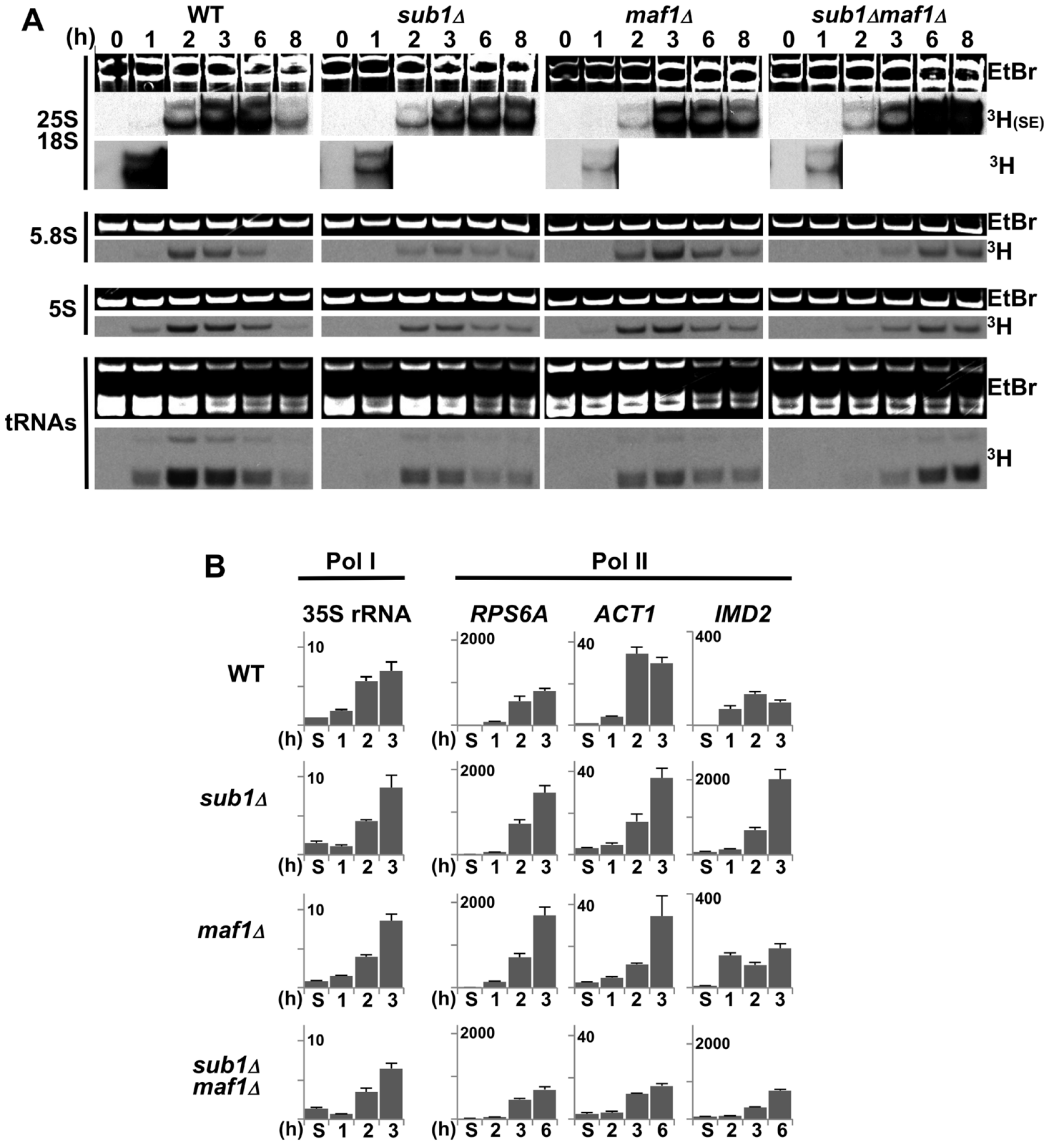
Reactivation of transcription upon exit from quiescence. (**A**) Day4 cultures of the indicated strains grown in SD4x were re-inoculated into fresh medium. RNA synthesis by Pol III (5S rRNA, tRNAs) or Pol I (25S, 18S, 5.8S rRNAs) was monitored over time by metabolic labelling with ^3^H-uracil (^3^H). For 25S and 18S rRNAs, a shorter exposure (SE) is shown. Total RNA loaded was visualized by staining with ethidium bromide (EtBr). (**B**) The expression of the 35S rRNA gene and of *RPS6A*, *ACT1* or *IMD2* genes, three of Sub1 genomic targets, in the indicated strains grown in YPD, at day5 (S) and after 1 to 6 h post-inoculation into fresh media was determined by real-time PCR. Expression levels were normalized to those of the 5S rRNA gene. Standard deviations were calculated from two independent preparations of cDNA. As expected from the repressive role of Sub1 on *IMD2* gene expression, a higher expression level of *IMD2* gene was detected in the absence of Sub1.

To obtain a global view of the reactivation of Pol III transcription, we next performed RNA metabolic labelling experiments that also allowed the visualization of Pol I transcription. Newly synthetized RNAs were monitored over time after the release of resting cells into fresh SD4x medium containing ^3^H-uracil (Fig. 3A). Although transcription is reduced but not totally abolished in stationary phase, no labelled signals could be observed in resting state probably due to the loss of uracil uptake [17]. In wild type, *sub1Δ* or *maf1Δ* strains, the reactivation of 5S rRNA or tRNAs transcription occurred within the 2 first hours post-inoculation with optimal levels of labelled signals observed at 2–3 h. The overall reactivation of Pol III transcription seemed to occur to a lesser extent in *sub1Δ* and *maf1Δ* cells as compared to wild type. At 6–8 h post inoculation, as also observed at an early stage in the growth cycle for Pol I transcription [18], Pol III transcription decreased in these three strains. In sharp contrast, the appearance of neo-synthetized Pol III transcripts was strongly delayed in the *sub1Δmaf1Δ* strain, with optimal levels of labelled signals reached only after 6–8 h post-inoculation, in good correlation with a longer delay in exiting quiescence (Fig. 1).

According to our previous genome-wide occupancy analysis [9], we wondered whether Sub1 could also play a role in the reactivation of Pol I and Pol II transcription. Metabolic labelling and RT-qPCR (Fig. 3) showed that the reactivation of Pol I transcription occurred similarly in all strains but to a lesser extent for the mutant strains as compared to wild type within the first two hours post inoculation. The appearance of the small 5.8S rRNA was delayed similarly to the 5S rRNA in the *sub1Δmaf1Δ* strain, probably due to the co-regulation of these two rRNAs [19]. Analysis of three Pol II-transcribed genomic targets of Sub1 [9], [11], [20] suggested that it may also interfere with the reactivation of *ACT1* and *IMD2* genes but not that of *RPS6A*. RT-qPCR further showed that the deletion of Maf1 resulted in a slightly delayed reactivation of *ACT1* (Fig. 3B). A strong delay for all three genes was observed in the *sub1Δmaf1Δ* cells with no reactivation at 2 h post-inoculation and optimal levels hardly reached at 6 h. These data indicate that both Sub1 and Maf1 may influence transcriptional reactivation and further investigation will be required to determine their role in this process.

**Figure 4:**
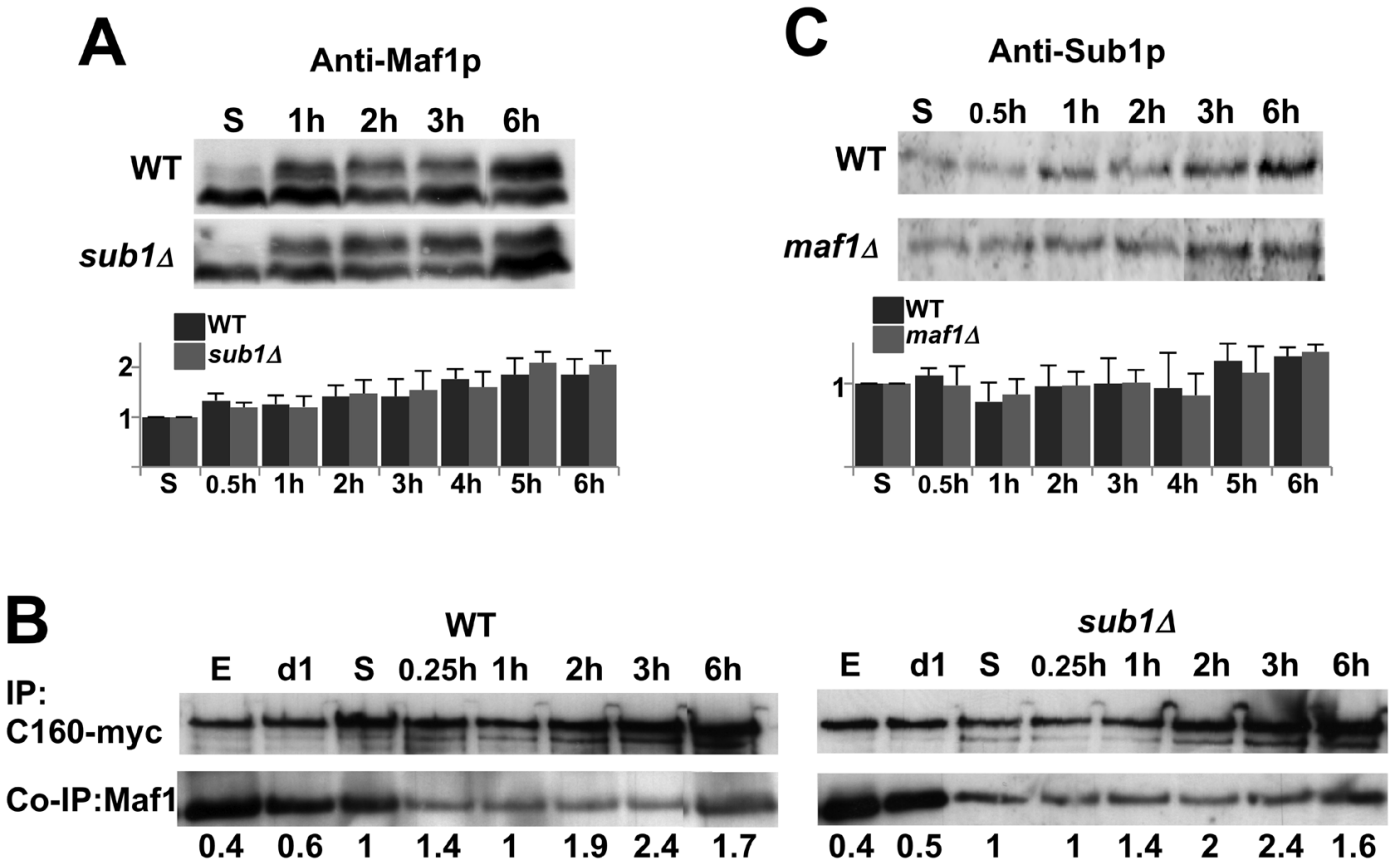
The difference in reactivating Pol III transcription is not due to an excess of Sub1 or Maf1. The amounts of Maf1 (**A**) or Sub1 (**C**) in the indicated strains grown in YPD at day5 or after 1 to 6 h post-inoculation into fresh media were analyzed by SDS-PAGE followed by immunoblotting using polyclonal antibodies as indicated. The signals obtained with anti-Maf1 antibodies correspond to hypo- (fast-migrating) or hyper- (slow migrating) phosphorylated forms of Maf1. The quantifications of Maf1 or Sub1 protein levels were normalized to a control protein band stained by Ponceau Red. Standard deviations were calculated from at least 3 independent experiments. (**B**) Cells expressing myc-tagged C160, the largest subunit of Pol III, in the presence (WT) or the absence of Sub1 (*sub1Δ*) *w*ere grown in YPD and collected either in exponential phase (E), at day1 (d1) and day5 (S) stationary phase or after 0.25 h to 6 h post-inoculation into fresh medium. Protein extracts were incubated with magnetic beads coated with anti-myc. The bound polypeptides were eluted and analyzed by SDS-PAGE followed by immunoblotting using anti-myc or polyclonal antibodies directed to Maf1. The quantified ratios of co-immunopurified Maf1/immunopurified C160-myc are indicated below each lane.

From all these experiments, we concluded that Sub1 plays a role in reactivating Pol III transcription at an early stage of the exit from resting. We figured out that the delay observed in the absence of Sub1 could be explained by an up-regulation of Maf1 repressing activity that could be due to the presence of higher amounts of dephosphorylated Maf1 or of repressing Maf1-Pol III complexes, or to a delay in Maf1 re-phosphorylation. However, no significant difference in the amounts or in the phosphorylation state of Maf1 could be detected between wild type and *sub1Δ* cells (Fig. 4A). Furthermore, a time course analysis of Maf1 interaction with Pol III by co-immunoprecipitation did not reveal either a stronger binding of Maf1 to Pol III in the absence of Sub1 (Fig. 4B). On the other hand, Pol III transcription could be reactivated sooner in the absence of Maf1 than in wild type cells but this effect was also not due to a difference in the amounts of Sub1 upon exit from quiescence (Fig. 4C). A slight increase of 1.7 fold in the levels of Sub1 protein was observed in both strains, in good correlation with the quite modest (2.5 fold) induction of *SUB1* mRNA upon these conditions [21].

### Sub1 and Maf1 play an opposite role on CLS

**Figure 5:**
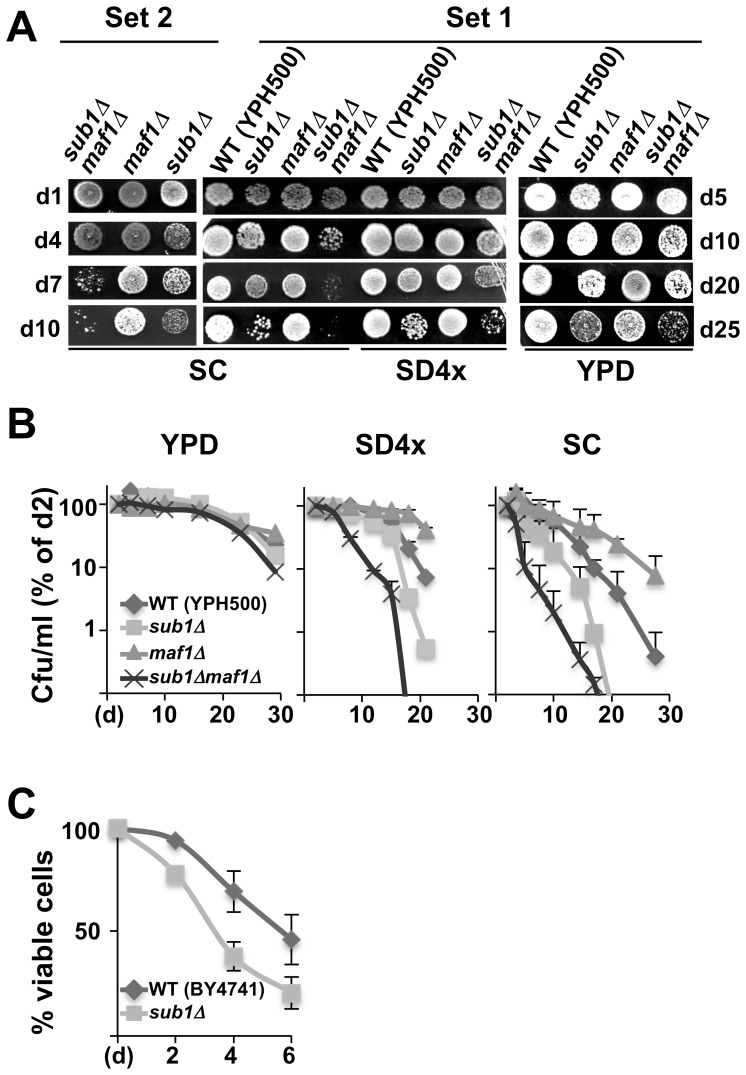
The decreased survival of quiescent cells deprived of Sub1 does not depend on strain genotypes or medium composition. (**A**) Two sets of the indicated strains generated in YPH500 with *sub1Δ::kanMX6* and *maf1Δ::ura3* deletion cassettes (Set 1) or with *sub1Δ::his3* and *maf1Δ::kanMX6* deletion cassettes (Set 2) were grown to stationary phase in YPD, SC or SD4x medium. Samples of cultures were spotted onto YPD plates at the indicated day (d1–d10 for SC or SD4x; d5–d25 for YPD) and the relative growth of the strains was observed after 4 days of incubation at 30°C. (**B**) Viability was determined over time by counting the number of colony forming units (Cfu) per volume of culture. Life-span curves represent means and standard deviations from at least two independent experiments. Note that life span curves obtained with the second set of strains in SC medium are similar to those in SD4x medium for the first set shown here. (**C**) Viability of the indicated strains grown in SC medium was determined over time by trypan blue exclusion assays. Viability curves represent means and standard deviations from three independent experiments.

Our trypan blue exclusion experiments (Fig. 1A) suggested that Sub1 and Maf1 could play an opposite role on CLS. To further explore these observations, we first assessed cell viability by spotting samples of quiescent cultures at different time. Since strain genotypes and medium composition could influence the survival of starving yeast (see for example refs [13], [22]), experiments were performed with resting cells in YPD, SD4x or SC medium, with two sets of null derivatives of Maf1 and Sub1 generated in YPH500 background using different deleting cassettes and with a *sub1Δ* mutant generated in BY4741 background. In all cases (Fig. 5A and 5C), cells deprived of Sub1 were defective in maintaining viability under non-growth conditions, i.e displayed a shortened CLS. To precisely assess the CLS of the strains, the proportion of living cells was measured over time by counting the number of colonies formed per unit volume of culture (Fig. 5B). Life span curves showed that the survival of *sub1Δ* or *sub1Δmaf1Δ* cells in SC medium was strongly reduced. In SD4x or YPD, all strains exhibited longer CLS but cells lacking Sub1 also lost viability prior to the wild type strain. In all media, *maf1Δ* cells displayed an extended CLS as compared to the wild type strain. These experiments showed that Sub1 and Maf1 play opposite roles on CLS.

### In the absence of Sub1, cells properly exit from exponential phase

The absence of Sub1 resulted in several phenotypes that were observed very rapidly after cells grown in SC medium exit from exponential phase, including a premature death from day2 (Figs. 1A and 5) and a flow cytometry profile at day3 not similar to wild type (Fig. 1C, lane 0h). We thus wondered whether the transition from exponential phase to quiescence occurred properly in cells deprived of Sub1. This transition is characterized by a number of physiological and biochemical changes [1], [2]. We investigated several of these traits to determine whether they were defective in the absence of Sub1.

**Figure 6:**
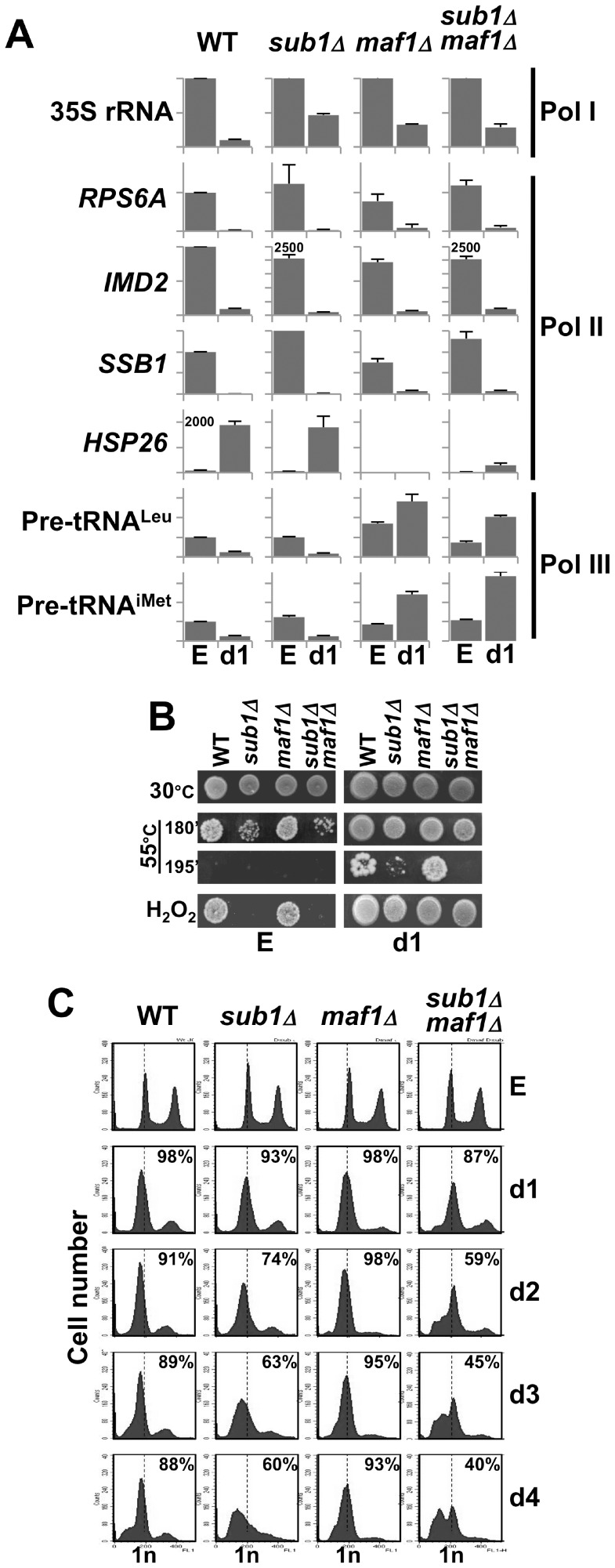
In the absence of Sub1, cells properly exit from exponential phase. (**A**) The expression of Pol I- (35S rRNA), Pol II- (*RPS6A*, *IMD2, SSB1, HSP26*) or Pol III- (Pre-tRNA^Leu^, pre-tRNA^iMet^) transcribed genes in the indicated strains grown in SC medium to exponential phase (E) or at day1 was determined by real-time PCR. Expression levels were normalized to those of the 5S rRNA gene and expression levels in the wild type strain grown to exponential phase was set to 100. Means and standard deviations from two independent preparations of cDNA are shown. (**B**) Samples of the indicated strains grown in SC medium to exponential phase (E) or at day1 were incubated at 55°C for the indicated time (in min) or in the presence of 25 mM H2O2 for 1 h and then spotted onto YPD plates and incubated at 30°C. (**C**) Changes in DNA content over time in samples of the indicated strains grown in SC medium to exponential phase (E) or at day1 to 4 were determined by flow cytometry. The percentage of viable cells determined by trypan blue staining at each time point is indicated. The percentage of cells in each phase was determined using the cell cycle modelling software ModFit LT 4.0 and is shown in S2 Fig.

First, a drastic decrease of transcription by all three RNA polymerases occurred in all strains during the transition from exponential phase to day1 (Fig. 6A) except for Pol III transcription repression that was delayed in cells deprived of Maf1 as described previously [23]. Analysis of Pol II-driven transcription showed that *sub1Δ*cells properly repressed *SSB1* and induced *HSP26* gene expression upon entry into resting as expected. Surprisingly, *HSP26* was not induced in the absence of Maf1.

Cells lacking Sub1 grown to exponential phase were sensitive to thermal or oxidative challenges but their resistance increased as expected at day1 (Fig. 6B). Flow cytometry was used to determine the proportion of cells arrested with a G1 DNA content from day1 to day4 in SC medium (Fig. 6C and S2 Fig.). The decreased height and narrowness of the 1n DNA peak observed in both *sub1Δ* and *sub1Δmaf1Δ* cells suggested that those cells displayed a less efficient G1 arrest than wild type. These cells rapidly suffered apoptotic DNA degradation as visualized by the appearance of sub-G1 DNA peaks correlated to an increase in the percentage of trypan blue stained cells. Strikingly, we noted that *maf1Δ* cells arrested more efficiently with a G1 content of DNA and displayed less rapid DNA degradation than wild type (Figs. 1C, 6C and S2 Fig.) in agreement with similar phenotypes observed for other long-lived mutants [24].

From these experiments we concluded that the transition from exponential phase to day1 occurred properly in cells deprived of Sub1. However, very rapidly these cells began to die suggesting that *SUB1* gene was necessary to maintain cell viability at a very early stage of the non-proliferative state.

### Loss of Sub1 results in an increased accumulation of reactive oxygen species

**Figure 7:**
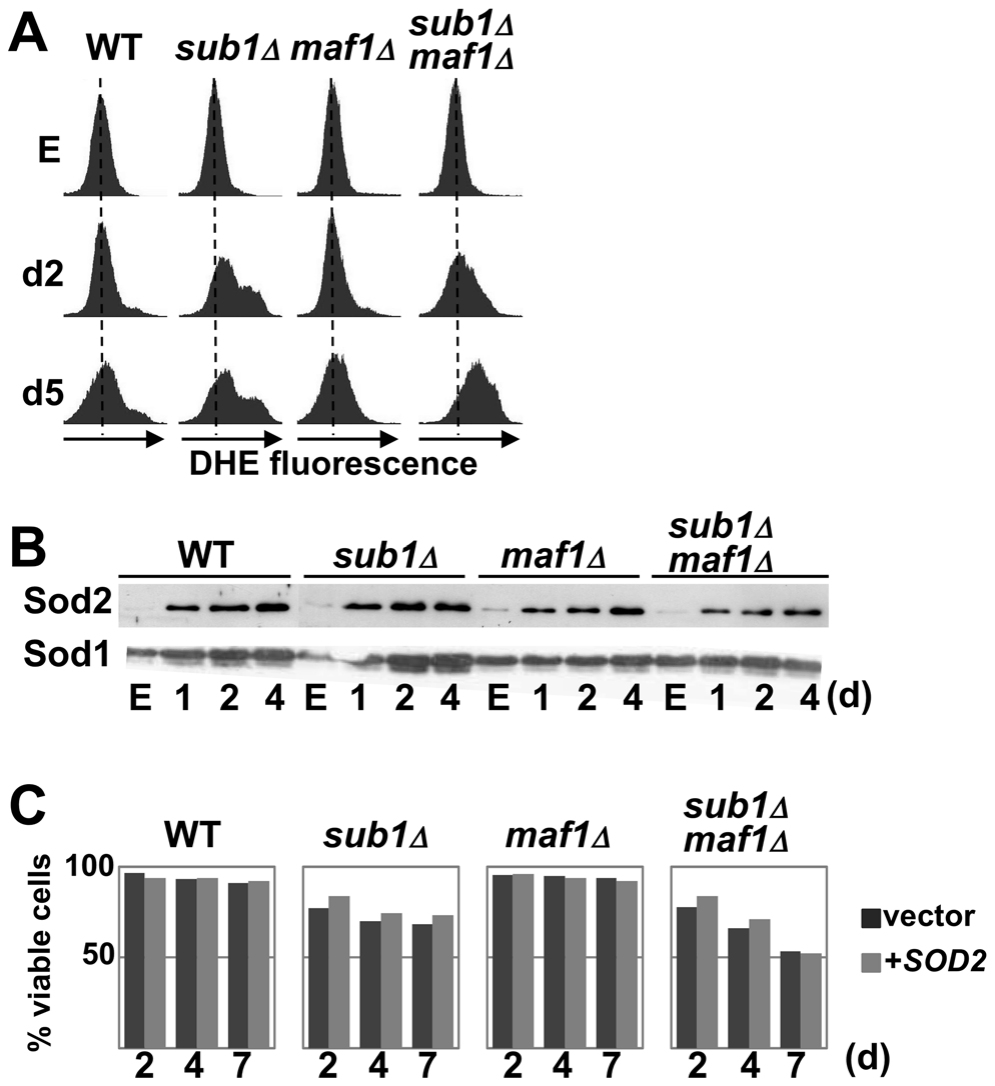
Loss of Sub1 results in an increased accumulation of reactive oxygen species. (**A**) Reactive oxygen species (ROS) levels in the indicated strains (Set 2) grown to exponential phase (E) or at day2 and day5 were measured using the ROS-sensitive fluorescent dye dihydroethidium (DHE) and flow cytometry. The appearance of DHE fluorescence signals on the right of the x axis is correlated to increasing ROS levels. (**B**) The levels of Sod2 and Sod1 in the indicated strains (Set 2) grown to exponential phase (E) and at day1, 2 or 4 were determined by Western blot analysis. (**C**) Viability in SC medium of the indicated strains (Set 2) transformed by a *URA3*-marked plasmid overexpressing *SOD2* gene was determined over time using trypan blue staining.

Many pathways encompassing a wide variety of functions have been shown to affect CLS in yeast [25] including the production of reactive oxygen species (ROS) by mitochondrial respiration that could be responsible for cellular damages and death. Two superoxide dismutases, Sod1 and Sod2 are critical enzymes for the detoxification of ROS. Interestingly, genetic interactions have been found between Sod1 and Sub1 [26], [27]. We thus explored the hypothesis that the shortened CLS observed in cells deprived of Sub1 could be linked to an increased accumulation of ROS. The intracellular ROS levels were measured by flow cytometry using the ROS-sensitive probe dihydroethidium (DHE). We found that ROS abundance in wild type and mutant cells was identical in exponential phase but diverged at day2 with *sub1Δ* and *sub1Δmaf1Δ* strains exhibiting significantly more ROS than wild-type and *maf1Δ* (Fig. 7A). Next, we analysed the steady-state abundance of Sod1 and Sod2 by Western blot and did not find any significant differences in the induction of these detoxification proteins in the absence of Sub1 (Fig. 7B). Furthermore, as shown in Fig. 7C, the overexpression of *SOD2* did not prevent the premature death of the strains deprived of Sub1. A higher accumulation of ROS in cells deprived of Sub1 could thus not be correlated to a defective induction of Sod2.

### Ras/PKA and Tor1/Sch9 pro-aging signalling networks are involved in Sub1 effect on cell viability

**Figure 8:**
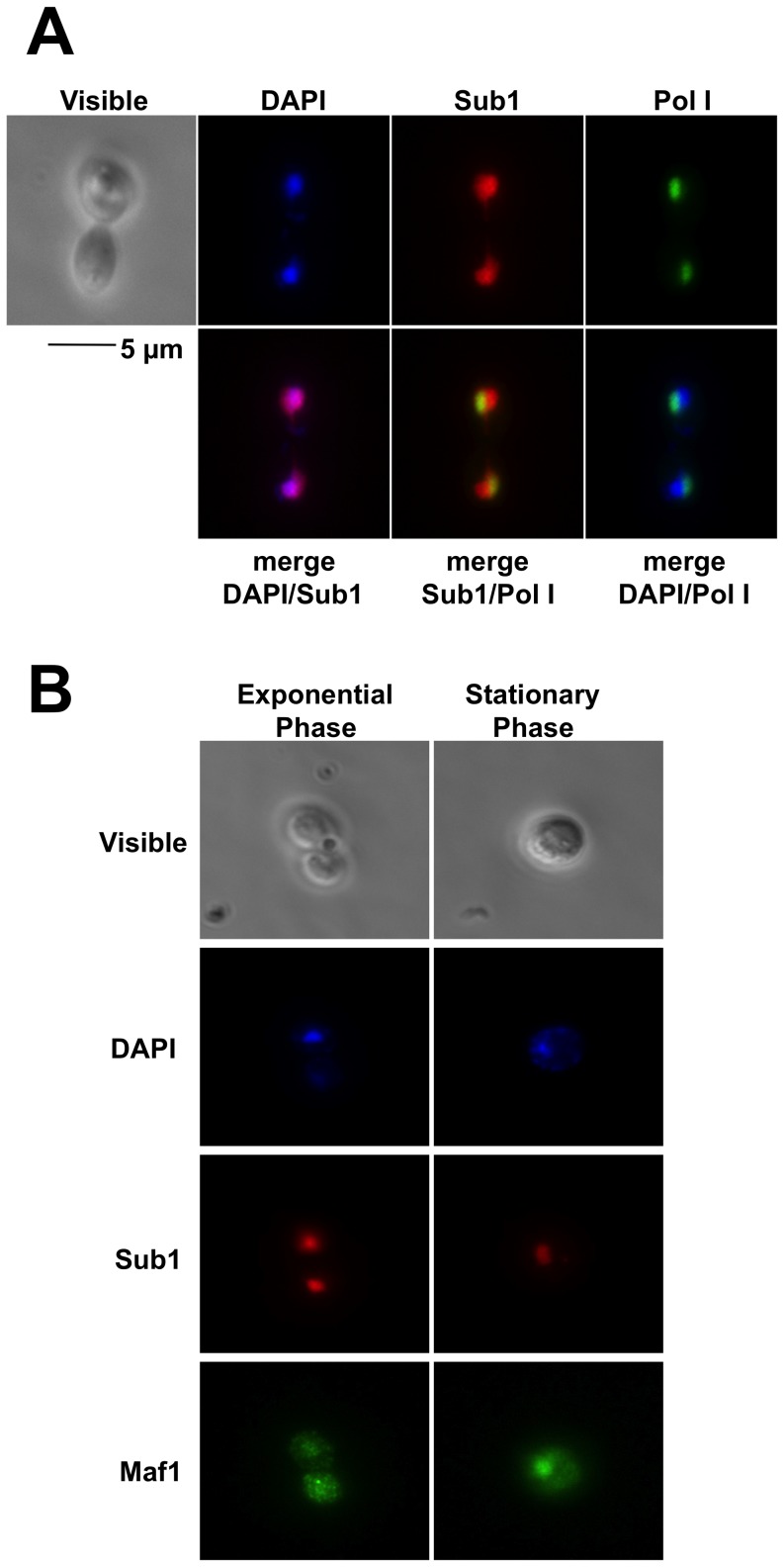
Intracellular localization of Sub1. Sub1 and Pol I (**A**) or Sub1 and Maf1 (**B**) were localized by immunofluorescence in exponentially growing cells in YPD or at day5 stationary phase, as indicated. The cells were analysed by phase contrast light microscopy (visible), by DNA staining (DAPI) and by immunofluorescence using antibodies specific to Sub1, Pol I or Maf1, as indicated. Overlays from the indicated combinations of images are shown.

The nutrient-sensing pathways controlled by Tor1/Sch9 and Ras/PKA regulate both Pol III transcription through Maf1 and quiescence through the Rim15 protein kinase [1]–[4], [28]. We thus wondered whether they could regulate cell survival and Pol III transcription through Sub1. The activity of most of the transcription factors including Rim15 and Maf1 that are downstream targets of these pathways is modulated by phosphorylation and cellular location [2]. Sub1 being a phosphoprotein [29], [30], we first analysed its intracellular localization by immunofluorescence in cells grown to exponential or stationary phase, using Maf1 as a control. In exponentially growing cells (Fig. 8A), the fluorescence signals corresponding to Sub1 were uniformly distributed throughout the nucleoplasm (visualized by DAPI staining) but also the nucleolus (visualized by Pol I signals). The nuclear locations of Sub1 were unchanged in stationary phase (Fig. 8B) suggesting that, in contrast to Maf1, Sub1 did not shuttle between the cytoplasm and the nucleus in response to growth conditions.

**Figure 9:**
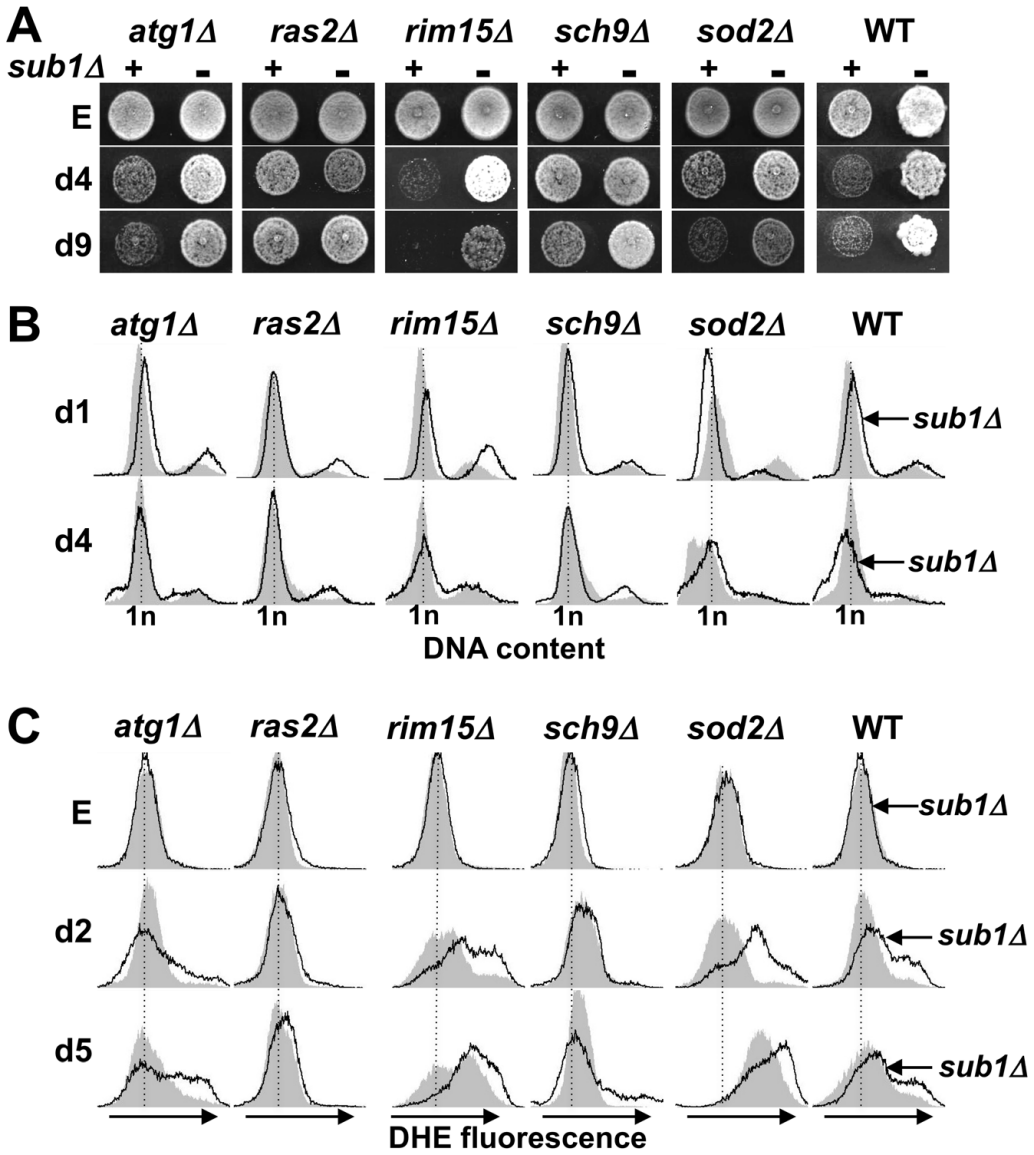
Inactivation of the Ras/PKA or Tor1/Sch9 signalling networks suppresses the effect of Sub1 deletion on quiescent cell survival. (**A**) Samples of the indicated strains, all uracil prototrophs, grown in SC medium to exponential phase (E) or at day5 and day9 were spotted onto YPD plates and incubated at 30°C. The growth of single (-) null mutants was compared to that of their combination with Sub1 deletion (Set 2, +). (**B**) The indicated strains were grown to stationary phase in SC medium. Changes in DNA content at day1 and day4 were determined by flow cytometry. The filled grey curves represent the profiles of single null mutants, the black lines that of their combination with Sub1 deletion. (**C**) ROS levels in the indicated strains grown to exponential phase (E) or at day2 and day5 were measured with DHE staining and flow cytometry. The filled grey curves represent the profiles of single null mutants, the black lines that of their combination with Sub1 deletion.

Next, we examined the relationships between Sub1 and components of these pathways by analysing several traits of quiescence in strains generated from combinations of *sub1Δ* with *sch9Δ*, *ras2Δ* or with null derivatives of downstream effectors of these two pathways controlling the quiescence program (*rim15Δ*), autophagy (*atg1Δ*) and detoxification of ROS (*sod2*Δ). Spotting assays of quiescent cells (Fig. 9A), flow cytometry analysis (Fig. 9B) and DHE fluorescence profiles (Fig. 9C) showed that the inactivation of the Ras/PKA pathway suppresses all phenotypes detected in *sub1Δ* strain. No premature death, no defect in the establishment of the G1 cell cycle arrest, no appearance of sub-G1 DNA peaks and no accumulation of ROS could be observed in the *ras2Δsub1Δ m*utant within the 9 first days of quiescence. The inactivation of the Tor1/Sch9 pathway similarly relieved *sub1Δ* deleterious effects during the 4–5 days of resting. These results suggested an upstream role of the Ras/PKA and to a lesser extent of the Tor1/Sch9 pathway in the regulation of the cell survival through Sub1. Since both pathways have overlapping effects on quiescence mainly through Rim15, we wondered whether Sub1 was a downstream effector of this kinase. This did not seem to be the case since the survival of *rim15Δsub1Δ w*as shortened as compared to the corresponding single mutant strains. The reduced viability of *rim15Δsub1Δ* or *sod2Δsub1Δ m*utant was probably due to an additive effect on ROS accumulation (Fig. 9C). In contrast, Atg1 deletion did not significantly change the phenotypes of *sub1Δ c*ells.

These data suggested that Ras/PKA and Tor1/Sch9 signalling networks are involved in Sub1 effect on cell viability.

## Discussion

Successful survival of starvation requires three distinct steps: induction of the quiescent state, maintenance of viability during quiescence and re-proliferation upon restoration of optimal growth conditions. In this study, we first demonstrate that Sub1 is involved in the transcriptional reactivation at an early stage of the exit from stationary phase. Second, loss of Sub1 impairs the capacity of the cells to properly establish a quiescent state and results in a premature death that is dependent on the Ras/PKA and Tor1/Sch9 signalling pathways. On the other hand, the inactivation of Maf1 extends long-term survival revealing a connection between Pol III transcription and yeast longevity. However, the phenotypes detected in *sub1Δmaf1Δ* cells and in both single null mutant do not correspond to classical synthetic genetic interactions and reveal that the connections between Sub1 and Maf1 are more complex than anticipated.

### Sub1 is required for an optimal reactivation of transcription upon recovery from starvation

Our data identify for the first time physiological conditions where Sub1 was needed for an optimal stimulation of Pol III transcription. A≈1 h delay to reach the optimal levels of Pol III transcription was detected in the absence of Sub1 when cells exit quiescence upon supply of fresh nutrients (Fig. 2). Preliminary experiments (Fig. 3) gave also the first evidence suggesting that Sub1 could be involved in the reactivation of Pol II transcription. Previous genome wide studies revealing a drastic decrease of Sub1 occupancy on its target genes in stationary phase [9] and a rapid up-regulation of its expression following the exit from resting [21] are in good agreement with a possible role of Sub1 in transcriptional reactivation. Further investigations will be required to confirm a direct role of Sub1 and to decipher the molecular mechanisms involved in these processes. However, in the case of Pol III-transcribed genes, since the high transcription rate observed in vivo probably relies on facilitated recycling pathway [31], the role of Sub1 in reinitiation [9] could explain the transcriptional effects depicted in this work. On the other hand, Sub1 was found to be associated with chromatin [32] and is acetylated [33]. It would be interesting to determine whether a more general effect of Sub1 on chromatin could contribute to the Pol II transcriptional reactivation. On the other hand, no significant difference in reactivating Pol I transcription was observed in the absence of Sub1 (Fig. 3). Sub1 binding to the 35S rRNA gene is controversial [9], [10]. However, in accordance with works that identified PC4 as a nucleolar protein [34], [35], we found that Sub1 was both a nucleoplasmic and a nucleolar protein (Fig. 8). A possible role of Sub1 in Pol I transcription is thus still an opened question.

Both Maf1 and Sub1 regulate Pol III transcription mainly through similar mechanisms. They have been shown to exert an opposite activity on the pre-initiation complex formation and in the reinitiation process [9], [36], [37] suggesting a simple counteracting effect of both effectors on Pol III transcription. However, the absence of Maf1 in *sub1Δ* cells strongly delayed the reactivation of Pol III transcription, an unexpected result that did not correlate with this basic model. Connections between Maf1 and Sub1 in regulating Pol III transcription are thus more complex than anticipated. The absence of Maf1 in *sub1Δ* cells also resulted in a reactivation of the three transcription machineries that may not occur at the same time (Figs. 2 and 3). This uncoordinated reactivation of transcription in *maf1Δsub1Δ* cells may result in an unbalanced supply of the ribosomal components and in a decreased capacity for protein synthesis that could explain the much longer time needed by this double mutant to re-proliferate.

### Sub1 is important to properly establish the quiescent state

In the absence of Sub1, cells properly exit the exponential phase and initiate the quiescent program. Transcription was rapidly shut down, stress response genes were induced and the cell resistance to temperature and oxidative stress increased (Fig. 6). However, s*ub1Δ* cells were impaired for proper G1 cell cycle arrest and they rapidly suffered apoptotic DNA degradation. The strongest phenotype in s*ub1Δ* cells entering quiescence was a rapid accumulation of ROS (Figs. 7 and 9). We could wonder whether ROS accumulation in s*ub1Δ* cells was due to a mitochondrial dysfunction or to a defect in ROS detoxification. No significant difference with the wild type strain could be detected in s*ub1Δ* upon entry into resting either in the resistance to oxidative stress (Fig. 6) or in the induction of Sod1 or Sod2 (Fig. 7B) suggesting that at least a part of the quiescence program dedicated to the cellular protection against oxidants was properly initiated in s*ub1Δ* cells. The increased ROS production in s*ub1Δ* cells may be responsible for their premature death as proposed by the mitochondrial theory of aging. Many studies have demonstrated that high levels of ROS represent one of the major factors that limit life span [25]. The correlation between lower ROS levels and increased viability observed in *ras2Δsub1Δ* or *sch9Δ*s*ub1Δ* cells, and higher ROS levels and reduced viability in *maf1Δsub1Δ rim15Δsub1Δ* or *sod2Δ*s*ub1Δ* cells (Fig. 9) may support this hypothesis. However, links between ROS levels and cell viability are complex and further investigations will be required to precisely determine the contribution of ROS levels in the premature death of *sub1Δ* cells.

The suppression effect of Ras2 and Sch9 deletion on *sub1Δ*defects strongly suggests that Sub1 may be part of an overlapping function of both Ras2/PKA and Tor1/Sch9 signalling pathways. Sub1 did not seem to be a direct target of PKA or Tor1/Sch9 kinases since it was not identified among PKA targets [38], its phosphorylation remained unchanged upon rapamycin treatment [30] and it did not shuttle between the cytoplasm and the nucleus in response to growth conditions (Fig. 8B). Sub1 did not seem either to be a downstream effector of Rim15 since *rim15Δsub1Δ* phenotypes were exacerbated as compared to the single null mutants (Fig. 9). Since Rim15-independent mechanisms involved in life span having already been described [39], another possibility could be that Sub1 acts in a parallel branch. We could not also rule out the fact that Sub1 may have a more global and complex role on quiescence due to its involvement in multiple cellular processes [6] including DNA damage, oxidative and replication stress that are also implicated in life span [25].

### Maf1, the key Pol III repressor, is also implicated in cell longevity

The deletion of Maf1, the key Pol III repressor extends cell longevity suggesting for the first time a connection between Pol III transcription and CLS. Both Ras/PKA and Tor1/Sch9 pathways are involved in the regulation of Pol III transcription mainly through Maf1 that is a direct target of both PKA and Sch9 kinase [40], [41]. Like *maf1Δ* cells, *ras2Δ* and *sch9Δ* display an extended CLS [42] suggesting that part of their long-lived phenotypes could result from their role in Pol III transcription.

Different works concerning Maf1 suggest putative mechanisms that may link Pol III transcription and cell longevity. First, as previously described [23], we noted an accumulation of tRNA precursors in *maf1Δ* cells upon entry into quiescence (Fig. 6). Similar defects were also observed when cells deprived of Maf1 were shifted from glucose to glycerol medium and were found to be due to a defective nuclear export of tRNAs [43]. Interestingly, impaired tRNA export contributes to the execution of the G1 checkpoint [44] and may explain the more efficient G1 arrest detected upon entry into quiescence in *maf1Δ* cells as compared to wild type (Figs. 1C and 6C). On the other hand, cytoplasmic tRNAs have been shown to accumulate in the nucleus upon entry into quiescence and to return to the cytoplasm in stationary phase [45]. Whether the nucleocytoplasmic distribution of tRNAs may play a role in CLS remains to be explored. We also wondered whether the higher levels of tRNAs in quiescent *maf1Δ* cells may play a role in aging through the Gcn4 transcription factor controlling the general amino acid control (GAAC) pathway. Interestingly, the regulation of GAAC through Gcn4 levels was found to affect CLS [46] and the accumulation of pre-tRNAs in *maf1Δ* cells was shown to increase Gcn4 levels [43]. However, the links between Pol III transcription, amino acid homeostasis and CLS seem to be complex. Our preliminary data showed a similar expression of Gcn4 in stationary phase in cells deprived of Maf1 or Sub1. Furthermore, *sub1Δmaf1Δ* cells also accumulated pre-tRNAs (Fig. 6) but, in contrast to *maf1Δ* cells, they displayed a strongly shortened CLS (Figs. 1A, 5 and 7C). Further experiments will thus be required to decipher the connection between these pathways and their implication in yeast longevity.

### Maf1 and Sub1, a connection more complex than anticipated

Trying to decipher the relationships between Sub1 and Maf1 that play an opposite role on Pol III transcription, we found that they also exert an opposite effect on CLS. Since our study indicates that Sub1, like Maf1, may be part of both Ras2/PKA and Tor1/Sch9 signalling pathways, we could wonder whether the observed phenotypes may be attributed to a role of Maf1 and Sub1 in two parallel pathways regulating both CLS and transcription. In this basic model, one could expect that turning off both genes would have a compensatory effect and would give a somewhat “wild-type” phenotype. However, in all our experiments under non-standard conditions, *sub1Δmaf1Δ ce*lls are always sicker or more affected than each of the single null mutants that strikingly exhibit opposite phenotypes as compared to wild type. This does not correspond to classical synthetic genetic interactions but to a single non-monotonic interaction as defined by Drees et al. [47] in which a mutant gene (*maf1Δ*) shows opposite effects in the wild-type background and the other mutant (*sub1Δ*) *b*ackground. The genetic-interaction inequality observed for Maf1 and Sub1 deletions could be represented by *maf1Δ*> Wild-type > *sub1Δ*> s*ub1Δmaf1Δ* (with > corresponding to greater than) and suggested than Maf1 and Sub1 act in at least two distinct functional pathways. In good accordance with this hypothesis, Sub1 is involved in several DNA-dependent processes including Pol II and Pol III transcription but is also important for cell survival upon several stress conditions. On the other hand, although the main known role of Maf1 is to repress Pol III transcription, recent studies suggest that Maf1 deletion may also affect other cellular processes [12], [48]. It would be interesting to determine which function(s) of Sub1 in any of these cellular processes renders its absence so deleterious in combination with Maf1 deletion, and vice-versa.

## Supporting Information

S1 Fig **The delay in exiting quiescence in the absence of Sub1 does not depend on media or strain genotype.** Optical density at 600 nm was monitored over time as a measure of regrowth of resting cells re-inoculated into fresh media. Growth curves represent an average from at least two independent experiments. (**A**) Set1 of strains generated in YPH500 background (day4 in SD4x). (**B**) Set2 of strains generated in YPH500 background with different deleting cassettes (day3 in SC). (**C**) Strains generated in BY4741 background (day3 in SC).
(TIF)

S2Fig **Cell cycle progression quantification.** (**A**) Quantification upon the exit from day3 stationary phase in SC medium corresponding to the flow cytometry analysis presented in Fig. 1C. (**B**) Quantification upon the entry into stationary phase corresponding to the flow cytometry analysis presented in Fig. 6C. The panels show the percentage of cells in sub-G1 peaks, or in G0/G1, S and G2/M phases of the cell cycle.
(TIF)
