# Supplementary figures and images for: Sub1 and Maf1, Two Effectors of RNA Polymerase III, Are Involved in the Yeast Quiescence Cycle
Source: PLoS One. 2014 Dec 22;9(12):e114587. doi: 10.1371/journal.pone.0114587 (PMC4273968; doi:10.1371/journal.pone.0114587)

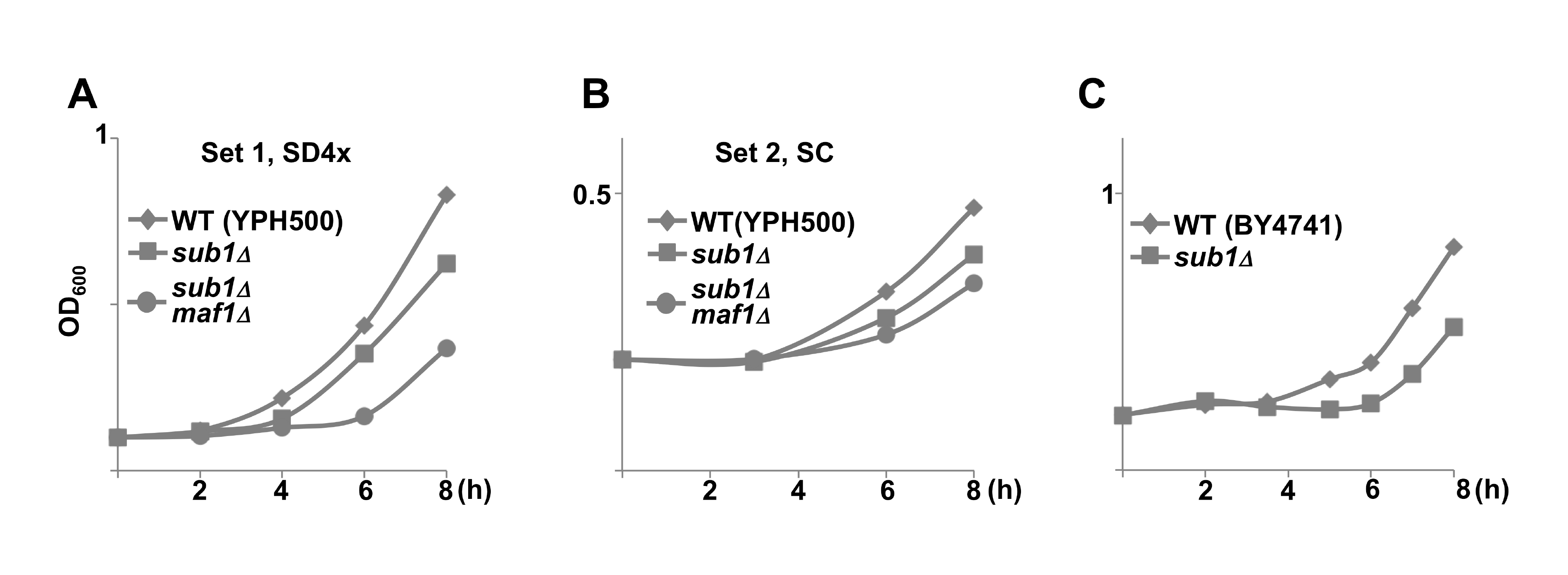

Supplement: S1 Fig — The delay in exiting quiescence in the absence of Sub1 does not depend on media or strain genotype. Optical density at 600 nm was monitored over time as a measure of regrowth of resting cells re-inoculated into fresh media. Growth curves represent an average from at least two independent experiments. (A) Set1 of strains generated in YPH500 background (day4 in SD4x). (B) Set2 of strains generated in YPH500 background with different deleting cassettes (day3 in SC). (C) Strains generated in BY4741 background (day3 in SC). (TIF) [file pone.0114587.s001.tif]

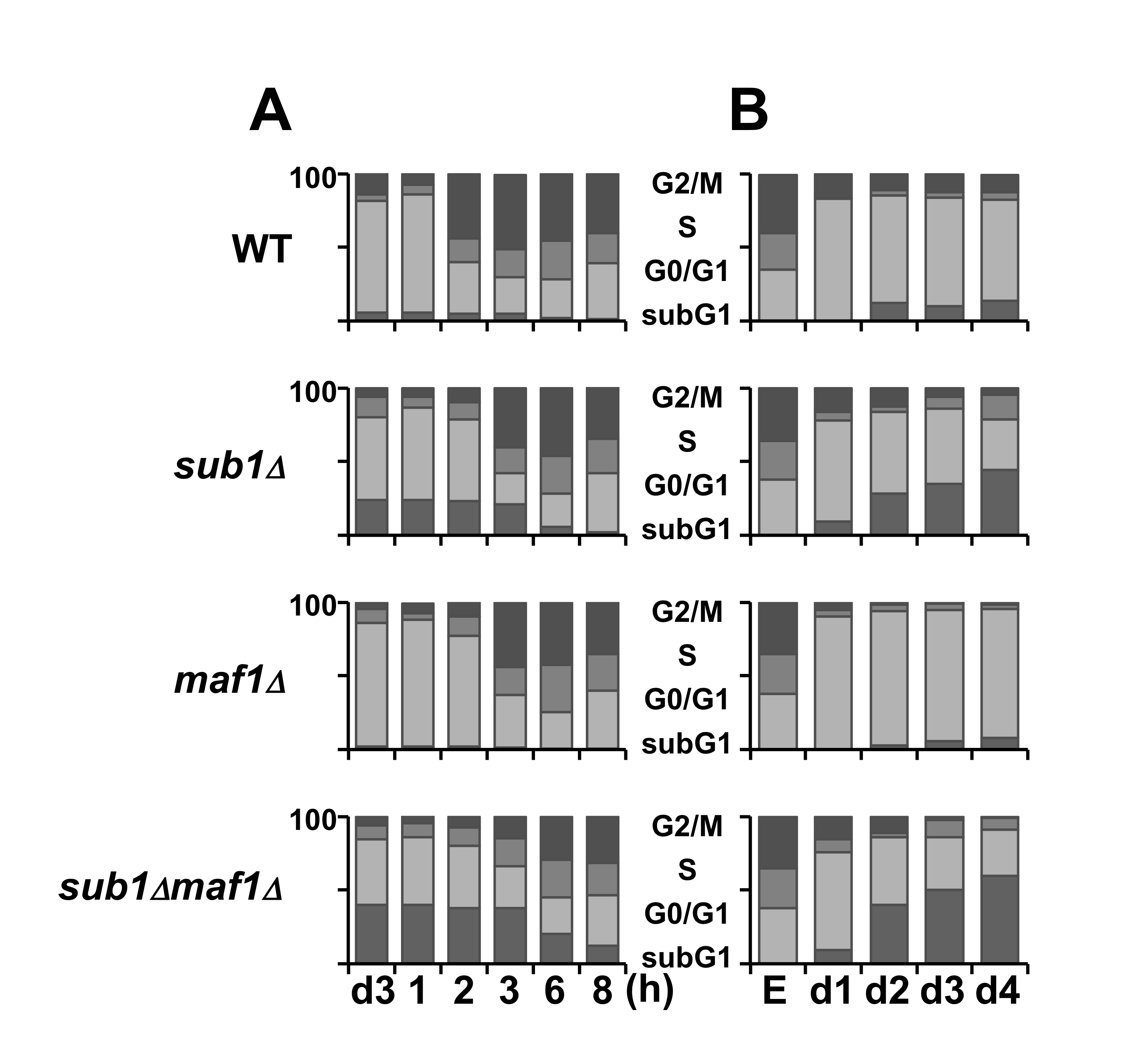

Supplement: S2Fig — Cell cycle progression quantification. (A) Quantification upon the exit from day3 stationary phase in SC medium corresponding to the flow cytometry analysis presented in Fig. 1C. (B) Quantification upon the entry into stationary phase corresponding to the flow cytometry analysis presented in Fig. 6C. The panels show the percentage of cells in sub-G1 peaks, or in G0/G1, S and G2/M phases of the cell cycle. (TIF) [file pone.0114587.s002.tif]
